# A systematic review of interventions addressing limited health literacy to improve asthma self-management

**DOI:** 10.7189/jogh.10.010428

**Published:** 2020-06

**Authors:** Hani Salim, Siti Nurkamilla Ramdzan, Sazlina Shariff Ghazali, Ping Yein Lee, Ingrid Young, Kirstie McClatchey, Hilary Pinnock, on behalf of the NIHR Global Health Research Unit on Respiratory Health (RESPIRE) collaborations

**Affiliations:** 1 NIHR Global Health Research Unit on Respiratory Health (RESPIRE), Usher Institute, The University of Edinburgh, United Kingdom; 2 Department of Family Medicine, Faculty of Medicine and Health Sciences, Universiti Putra Malaysia, Malaysia; 3 Department of Primary Care Medicine, Faculty of Medicine, University of Malaya, Malaysia; 4 Centre for Biomedicine, Self and Society, Usher Institute, The University of Edinburgh, United Kingdom; 5 Asthma UK Centre for Applied Research, Usher Institute, The University of Edinburgh, United Kingdom

## Abstract

**Background:**

Supported asthma self-management improves health outcomes. However, people with limited health literacy, especially in lower-middle-income countries (LMICs), may need tailored interventions to enable them to realise the benefits. We aimed to assess the clinical effectiveness of asthma self-management interventions targeted at people with limited health literacy and to identify strategies associated with effective programmes.

**Methods:**

Following Cochrane methodology, we searched ten databases (January 1990 – June 2018; updated October 2019), without language restriction. We included controlled experimental studies whose interventions targeted health literacy to improve asthma self-management. Selection of papers, extraction of data and quality assessment were done independently by two reviewers. The primary outcomes were clinical (asthma control) and implementation (adoption/adherence to intervention). Analysis was narrative.

**Results:**

We screened 4318 titles and abstracts, reviewed 52 full-texts and included five trials. One trial was conducted in a LMIC. Risk of bias was low in one trial and high in the other four studies. Clinical outcomes were reported in two trials, both at high risk of bias: one of which reported a reduction in unscheduled care (number of visits in 6-month (SD); Intervention:0.9 (1.2) vs Control:1.8 (2.4), *P* = 0.001); the other showed no effect. None reported uptake or adherence to the intervention. Behavioural change strategies typically focused on improving an individual’s psychological and physical capacity to enact behaviour (eg, targeting asthma-related knowledge or comprehension). Only two interventions also targeted motivation; none sought to improve opportunity. Less than half of the interventions used specific self-management strategies (eg, written asthma action plan) with tailoring to limited health literacy status. Different approaches (eg, video-based and pictorial action plans) were used to provide education.

**Conclusions:**

The paucity of studies and diversity of the interventions to support people with limited health literacy to self-manage their asthma meant that the impact on health outcomes remains unclear. Given the proportion of the global population who have limited health literacy skills, this is a research priority.

**Protocol registration:**

PROSPERO CRD 42018118974

Asthma self-management support, including written action plans and regular reviews by health care professionals, improves health outcomes [1-4]. Systematic reviews and guidelines highlight that cultural or age-related tailoring enables the successful implementation of supported self-management, although rarely specify tailoring for people with limited health literacy. This is a significant oversight, as health literacy is a problem globally [5], and a particular challenge in low and middle-income countries (LMICs). There is thus a need to address the challenges of providing support for people with limited health literacy [6,7].

A review of health literacy definitions by Sørensen et al. (2012), describes health literacy as people’s knowledge, motivation and competence to assess, understand, appraise and apply health information to make decisions on health care, disease prevention and health promotion throughout the life course (**Table 1**) [8]. These skills are essential for individuals to respond to the demands of managing a variable condition such as asthma, including adherence to medication, adjusting treatment and/or deciding to seek advice in the event of deterioration. Health literacy is not linearly related to health outcomes but influences three aspects of health care behaviour: access and utilisation of health services, patient-provider interactions and self-management [12].

**Table 1:** Definition of terms

Terms	Definition	Operational definitions
Self-management	The tasks that individuals must undertake to live with one or more chronic conditions. These tasks include having the confidence to deal with medical management, role management and emotional management of their conditions [4].	We included asthma self-management interventions including components described in the taxonomy of self-management support by Pearce et al. [3]:
		a) Direct components (delivered directly to patients and/or carers) such as education, action plans and practical support with adherence.
		b) Indirect components: health or social care professional level (delivered to individual health or social care professionals) such as equipment, feedback and review.
		c) Indirect components: delivered at an organisational level such as prompts using paper or electronic reminders.
Health literacy	Health literacy is linked to literacy and entails people's knowledge, motivation and competencies to access, understand, appraise, and apply health information in order to make judgments and take decisions in everyday life concerning health care, disease prevention and health promotion to maintain or improve quality of life during the life course [8].	We included interventions that:
		Measured the health literacy level of the study population using a validated tool, and if 40% and more of the participants had limited health literacy.
		Studied a population with published evidence of a high prevalence of limited health literacy. Examples were: immigrants, ethnic minorities, ‘illiterate women’ [9].
		We also included any interventional designs which explicitly aimed to improve health literacy using techniques described by Sheridan et al. [10]:
		a) Presenting written information differently (eg, essential information first).
		b) Presenting numerical information differently (eg, the highest number is better).
		c) Using icons, symbols and graphs.
		d) Presenting information pitched at a lower literacy level (eg, primary school comprehension).
		e) Use of videos.
		f) Literacy training for patients and physicians.
		g) Implementing comprehension skills to enable self-care.
Severe asthma attacks	Deterioration of asthma control that requires urgent action on the part of the patient and physician to prevent a serious outcome, such as hospitalisation or death from asthma [11].	Relevant actions included commencing a course of oral steroids, emergency admission

Two previous systematic reviews have looked at self-management interventions for people with limited health literacy in long-term health conditions [10,13]. One review included 38 studies, but only 22 were randomised trials, and none addressed self-management interventions in asthma [10]. The other defined the target population as people from low socio-economic groups, assuming that these populations had limited self-literacy [13]. Neither, therefore, specifically addressed supported management for people with limited health literacy in asthma. We this aimed to systematically search and synthesise the trial evidence for asthma self-management interventions targeted at people with limited health literacy, in order to assess their clinical effectiveness and to identify the behaviour change strategies that were associated with effective programmes [14].

## METHODS

This review is registered with the PROSPERO database (registration number: CRD 42018118974). Details of the systematic review protocol have been published [14] with salient points described here. We followed the procedures described in the Cochrane Handbook for Systematic Reviews of Interventions [15].

### Deviations from the published protocol

To be inclusive of data from LMICs, we intended to search the African Index Medicus, Africa Portal Digital Library; Index Medicus for the Southeast Asia Region; IndMed; Latin American and Caribbean Health Science Literature Database (LILACS). However, we decided to omit these after a scoping exercise revealed a lack of controlled trials in these databases, and we considered it was very unlikely that any publications would fulfil our inclusion criteria.

We intended to use the Grading of Recommendations Assessment Development and Evaluation (GRADE) to assess the weight of evidence of the reported outcomes from the included studies [16]. However, there was too much missing information to use GRADE. We have, therefore not presented the GRADE assessment in the paper (see Table S1 in the **Online Supplementary Document**).

### Search strategy

We searched 10 electronic databases (**Table 2**). The search strategy used medical subject headings (MeSH) and text words related to health literacy, asthma, self-management and controlled trial. The initial search (January 1990 to June 2018) was updated in October 2019. We conducted forward citation on included studies and contacted experts in the field to identify related trials. We did not perform manual searches as no journal(s) emerged as having a particular interest in this topic. There was no language restriction, though we did not find any non-English publications. We searched the databases using PICOS criteria (**Table 2**). We used the definitions in **Table 1** to confirm eligibility.

**Table 2:** PICOS table and operational definitions

PICOS	Descriptions and definitions	Operational definitions
**Population**	Physician-diagnosed asthma or their parents/carers.	Any age: children, adolescent, adults and /or the elderly
**Intervention**	Asthma self-management targeted at participants with limited health literacy level, noting how the authors’ definitions	See **Table 1** for our definitions.
		We included interventions which trained health care practitioners to support self-management in people with limited health literacy if the outcomes included the impact on the patient.
**Comparator**	Usual care or alternative interventions	For example: lower intensity self-management strategies, or interventions not targeting health literacy.
**Outcomes**	**Primary health outcomes**	Asthma control measured by a validated questionnaire such as the Asthma Control Questionnaire [17] or Asthma Control Test [18]).
	Based on the European Respiratory Society/American Thoracic Society ERS/ATS Task Force report [11] health outcomes were:	Asthma attacks were defined in line with the ERS/ATS definition of ‘severe asthma exacerbations’ (see **Table 1**):
	• Current asthma control (eg, control questionnaires)	
	• Asthma attacks (eg, number of severe attacks, steroid courses, emergency department visits, hospitalisations).	
	**Primary implementation outcomes**	
	• Adoption of the intervention	
	• Adherence to intervention	
	**Secondary outcomes:**	Examples of adoption/adherence included proportion of participants taking up the intervention, provided with, or frequency of usage of, an action plan).
	Self-efficacy, activation, empowerment, health literacy.	Secondary outcomes were intermediate measures known to reflect self-management skills, or other evidence of impact.
	Improvement in knowledge, Correct inhaler use.	
	Cost-effectiveness, fidelity and sustainability.	
**Setting**	Any clinical or community-based setting in any country (developed or developing nations).	
**Study design**	Controlled experimental studies: Randomised controlled trials (RCTs), controlled clinical trials, controlled before-and-after studies and interrupted time-series designs.	
**Database searched**	MEDLINE: EMBASE: CINAHL Plus: PsycINFO: AMED: BNI: Cochrane Library: Database of Abstracts of Reviews of Effects, Cochrane Database of Systematic Reviews (CDSR) and Cochrane Central Register of Controlled Trials (CENTRAL); Web of Science Core Collection; ScienceDirect; Global Health.	

### Study selection and data extraction

After training and quality control, two authors (HS and SNR) independently screened the de-duplicated titles and abstracts. We obtained the full text of potentially relevant studies, and both reviewers independently assessed for eligibility. Disagreements or uncertainties at any stage were resolved by discussion within the team (HP, IY, SGS or PYL).

Studies which had multiple publications (eg, a protocol, trial findings, process evaluations, qualitative studies, translations) were treated as one study, and reference made to the different publications.

We piloted a data extraction form adapted from the Effective Practice and Organisation of Care (EPOC) recommendations for describing interventions [19] and the Template for Intervention Description and Replication (TIDieR) checklist [20]. Two reviewers (HS and SNR) independently extracted data. We contacted authors for any information which was not found within the included paper(s).

### Quality assessment

We used the Cochrane Risk of Bias tool [15], and the guidance from the EPOC group [19], to assess selection, performance, detection, attrition, reporting and other potential sources of bias [15]. The risk of bias for each domain was classified as ‘low', ‘high' or ‘unclear' based on the information available [15]. We generated ‘risk of bias‘ summary graphs and figures using Review Manager 5.3 [21].

### Outcomes

Outcomes are described in **Table 2**. We were primarily interested in health outcomes (eg, asthma control; acute attacks) and implementation outcomes (eg, adoption of intervention). Secondary outcomes included intermediate self-management measures (eg, knowledge improvement), health literacy outcomes and impact indicators (eg, cost-effectiveness).

### Data synthesis.

We conducted two analyses to answer the two objectives of our systematic review. First, we considered the effectiveness of asthma self-management interventions which addressed health literacy needs compared with the control group. From scoping work, we anticipated that the studies included in this review would vary substantially in design, target populations, outcomes measured and duration of follow-up precluding meta-analysis. We, therefore, conducted a narrative synthesis of the data.

**Figure 1:**
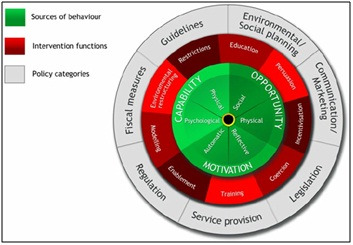
The Behaviour Change Wheel (BCW). Reproduced with permission from BioMed Central Ltd [22].

Second, we described and characterised the included interventions using the Behaviour Change Wheel (BCW) framework (**Figure 1**), which provides a systematic way to describe and characterise the techniques used in the interventions in this review [22,23]. The BCW has three layers; its core components consist of the COM-B system (Capability, Opportunity and Motivation); interactions between these components determine Behaviour [22]. Capability is the individual’s psychological and physical capacity to engage in the behaviour. It includes having the required knowledge and skills. Motivation is defined as processes that contribute towards both reflective and automatic mechanisms that activate or inhibit behaviour. Opportunity includes aspects of the physical and social environment that lie outside the individual that prompt or make behaviour possible. The second layer of the BCW describes the nine functions of interventions that are designed to change behaviour. The intervention functions are; education, persuasion, coercion, training, enablement, modelling, environmental restructuring and restrictions. The third layer of the BCW identifies seven types of policies (eg, legislation, fiscal measures, etc.) that can be applied to deliver these intervention functions [22].

It is proposed that specific intervention functions are likely to influence change in the specific target behaviour. This underpins a matrix (**Table 3**), produced through a consensus exercise amongst behaviour change experts [22], that enables gaps in intervention functions required to impact on the three core components (capability, opportunity and motivation) that govern behaviour change [22].

**Table 3:** Mapping of core components of behaviour and intervention functions used in the included studies*

Core components of behaviour, COM-B model		Intervention functions								
**Education**	**Persuasion**	**Incentivisation**	**Coercion**	**Training**	**Restriction**	**Environmental restructuring**	**Modelling**	**Enablement**
**Capability**	**Physical**					**Macy et al. [**24**], Poureslami et al. [**25**], Ozyigit et al. [**26**] †**				†
	**psychological**	**Macy et al. [**24**], Poureslami et al. [**25**], Ozyigit et al. [**26**]†**				†				**Yin et al. [**27**], Ozyigit et al. [**26**], Apter et al. [**28**]†**
**Opportunity**	**physical**					†	†	†		†
	**social**						†	†	†	†
**Motivation**	**automatic**		†	†	†	†		†	†	†
	**reflective**	†	**Apter et al. [**28**], Poureslami et al. [**25**]†**	†	†					

*This matrix links the core components that drive behaviour (COM-B) to the intervention functions [22,23]. The matrix and the marked (†) boxes were identified through a consensus exercise by a group of experts [22,23]. The marked (†) boxes indicate where the consensus group considered that intervention functions linked to the COM-B model. For example, (1) physical capability can be achieved through physical skill development which focuses on training and enabling interventions; (2) psychological capability can be achieved through education, training and enabling interventions; (3) reflective motivation can be achieved through education, persuasion, incentivisation, and coercion. (4) physical and social opportunity can be achieved through intervention functions including training, restriction, environmental restructuring, enablement, and modelling. In the matrix, we plotted the interventions included in this review according to their respective core components of behaviour and intervention function (see Table S2 in the **Online Supplementary Document** for the mapping exercise) through the following process: 1. The mapping process was conducted independently by two reviewers, HS and KM; 2. We identified the BCW core components of behaviour and the intervention functions used in each included study; 3. Working together, we plotted our findings within the matrix; 4. The studies included in this review are placed in the marked (†) boxes based on the targeted behaviours and the intervention functions used in each intervention; 5. Marked (†) boxes without studies are intervention functions that were not used in included studies and thus represent gaps that could be utilised in future interventions.

We plotted the components of interventions in this review onto the matrix. In the mapping process, which was completed independently by two reviewers (HS and KM), we first identified the core components of behaviour that were targeted, and also the intervention functions used in each included study. Through a consensus approach (see Table S2 in the **Online Supplementary Document**), we plotted our findings within the matrix (**Table 3**).

## RESULTS

**Figure 2:**
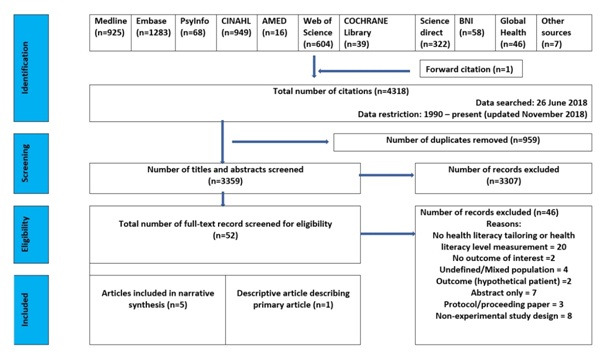
PRISMA flowchart.

The selection process is illustrated in the PRISMA diagram (**Figure 2**). From 3359 papers, we selected six papers describing five randomised control trials [24-28] (the sixth paper described the development of the intervention[29]). The studies included a total of 731 participants in the intervention groups and 561 participants in the control groups [24-28].

### Characteristics of included studies

The randomised control trials were conducted from 2011 to 2017; four studies were conducted in high-income countries [24,25,27,28] (three in the United States (US); one in Canada) and one in Turkey (a middle-income country) [26]. **Table 4** summarises population characteristics (see detailed descriptions in Table S3 in the **Online Supplementary Document**).

**Table 4:** Summary of impact of clinical and process outcomes categorised by health literacy status of the population.

Citation, design, Follow-up (FU), n, ethnicity, age, asthma control, Risk of bias	Intervention summary	Reported outcomes (* indicates the trial’s primary outcome (if stated))	Interpretation of effectiveness
**Health literacy status of the population:** All population with limited health literacy (assumption based on quantitative & qualitative evidence in the literature).			
**Poureslami 2012 [**25**]**			
Canada, RCT, FU: 3mo, one centre, 85 adults, minority population (Chinese & Punjabi); at least 21 y-old, baseline asthma control:	Participants watched videos on asthma knowledge and/or community’s cultural views on asthma.	**Asthma control**	No relevant outcome.
**The overall risk of bias: High risk**	Study groups:	**Unscheduled care**	No relevant outcome.
	I^K^: Knowledge video		
	I^C^: Community video		
	I^K+C^: Both videos		
	C: pictorial leaflet	* Inhaler technique	No between group comparison “Proper use of inhalers improved significantly among all experimental groups over time P< 0.001”.
		*Inhaler technique*: Within group comparison of inhaler technique score at baseline and 3mo: Mean (SD) mean difference; MD (95%CI)	**Insufficient details to gauge effectiveness.**
		I^K^: B 4.0 (2.1) vs 3mo 5.9 (2.0); MD 2.71 (1.35 to 4.06)	
		I^C^: B 4.5 (2.0) vs 3mo 6.8 (2.0); MD 1.95 (0.99 to 2.91)	
		I^K+C^: B 3.9 (2.1) vs 3mo 6.8 (1.6); MD 1.53 (0.66 to 2.40)	
		C: B 4.8 (2.3) vs 3mo 6.6 (1.4); MD 1.05 (-0.10 to 2.20)	
		*Knowledge:	No between group comparison. “There was a significant difference in mean scores in the improvement of knowledge in asthma symptoms, triggers and the understanding of physician instructions on medication use between intervention groups and control” *P* < 0.05.
		*Understanding physician instructions on medication use*: Within group comparison of inhaler technique score at baseline and 3mo; Mean (SD), MD (95%CI).	**Insufficient details to gauge effectiveness.**
		I^K^: B 0.8 (0.6) vs 3mo 1.4 (0.8) MD 0.53 (0.12 to 0.94)	
		I^C^ 2: B 1.2 (0.9) vs 3mo 1.7 (0.9) MD 0.38 (-0.06 to 0.82)	
		I^K+C^: B 1.7 (0.8) vs 3mo 1.8 (0.6) MD 0.24 (-0.19 to 0.66)	
		C: B 1.6 (1.1) vs 3mo 1.7 (0.8) MD 0.35 (-0.22 to 0.92)	
**Ozyigit 2017 [**26**]**			
Turkey, RCT, FU: 6 mo, one centre, 34 female adults, 18 to 55 y-old, baseline asthma control: Uncontrolled.	Participants received pictorial asthma action plans (PAAP) and education materials. The PAAP was previously used among people with low levels of education and asthma.	*Asthma control	There was no significant difference between intervention and control for asthma control and QoL.
**The overall risk of bias: High risk**	Study groups:	Between group difference at 6mo in asthma control test, ACT; Mean (SD)	**Consistently shown as no effect.**
	I: Pictorial AAP + educational materials	I: 24.0 (1.0) vs C:23.3 (1.3) *P* = 0.07	
	C: educational materials	**Health-related quality of life (QoL)**	
		Between group difference at 6mo in St. George Respiratory Questionnaire, SGRQ; Mean (SD).	
		I: 53.7 (7.5) vs C:50.3 (7.8), *P* = 0.21	
		*Unscheduled care	The significant difference was seen between control and intervention group for number of emergency visits during the study period.
		Between group difference in number of visits to the emergency department over the 6mo study period; Mean (SD)	**Consistently shown as positive effect.**
		I: 0.9 (1.2) vs C:1.8 (2.4), *P* = 0.001	
**Health literacy status of the population** Majority limited health literacy, n (%)			
**Yin 2017 [**27**]**			
US, RCT, FU: post intervention	Participants (carer of child with asthma) received low-literacy, plain language, pictogram-, and photograph-based asthma action plans. Parents were asked what they knew about medication used in managing chronic asthma from their understanding of the pictorial asthma action plan (PAAP).	**Asthma control**	No relevant outcome.
Paediatric OPD, carers of 217 children, majority Hispanics; mean age 35.5 y (8.3), proportion with limited health literacy I: 74% vs C: 65%aseline asthma control: mild - moderate severe.			
**The overall risk of bias: Low risk**	Groups:	**Unscheduled care**	No relevant outcome.
	I: pictorial PAAP	**Perceived ease of use**	No significant between-group difference in proportion expressing trouble reading the allocated PAAP, though when shown both PAAPs 79% considered the low-literacy PAAP easier to understand.
	C: standard PAAP	*Perceived ease of use (PAAP):* Between group comparison of proportion expressing trouble reading the allocated PAAP; n (%).	**Illustrated as consistently no effect**
		I: 93 (85%) vs 93 (88%), *P* = 0.7	
		*Knowledge	There was a significant between group difference in the knowledge of which maintenance medications to give and knowledge of spacer use, but not in the knowledge of appropriate emergency response.
		*Green/yellow zone knowledge*	**Overall, has shown positive effect but not all outcomes are consistent.**
		Between group comparison of proportion of carers making errors in the knowledge of which medications to give; n (%)	
		I: 63 (63.0) vs 75(77.3) *P* = 0.03	
		Between group comparison of proportion of carers making errors in knowledge of need for spacer use; n(%)	
		I: 14 (14.0) vs 48 (51.1) *P* < 0.001	
		*Red zone knowledge*	
		Between group comparison of proportion of carers making errors in knowledge of appropriate emergency response; n (%)	
		I: 47 (43.1) vs 52 (48.1) *P* = 0.5	
**Apter 2011 [**28**]**			
RCT, FU: 6mo, two centres, 333 adults, majority African American, more than 18 y-old, majority adequate health literacy, mean (SD)	Participants received 4 steps problem-solving intervention in the aspect of asthma and its management. The intervention allows critical evaluations of needs and concerns about asthma and its management & educate participants on how to overcome these problems.	*Asthma control	No between group comparison for quality of life and FEV1 predicted.
I:31.1 (7.6) vs C:31.4 (7), baseline asthma control: Mild			
**The overall risk of bias: High risk**	Groups	*Asthma control based on asthma control questionnaire, ACQ score:* Within group comparison from baseline, 3mo and 6mo; mean (SD)	“Asthma control improved significantly (P =0.002) for both groups, but there was no significant statistical or clinical difference between groups.”
	I: Problem solving sessions (PS) + Asthma education (AE)	I: B 1.7 (1.1) vs 3mo 1.6 (1.3) vs 6mo 1.5 (1.2)	“FEV1 percent predicted and quality of life improved from baseline: (*P* = 0.01) and (*P* < 0.0001).”
	C: Asthma education (AE)	C: B 1.7 (1.1) vs 3mo 1.5 (1.1) vs 6mo 1.3 (1.1)	**There was no effect for asthma control. Insufficient details to gauge effectiveness for quality of life & FEV1 predicted.**
		*Quality of life based on asthma QoL score*: Within group comparison from baseline, 3m and 6m; mean (SD)	
		I: B 4.0 (1.4) vs 3mo 4.7 (1.4) vs 6mo 4.7 (1.3)	
		C: B 4.1 (1.4) vs 3mo 4.8 (1.4) vs 6mo 4.8 (1.4)	
		*Forced expiratory volume, FEV1% predicted:* Within group comparison from baseline, 3m and 6m: mean (SD)	
		I: B 4.0 (1.4) vs 3m 4.7 (1.4) vs 6m 4.7 (1.3)	
		C: B 4.1 (1.4) vs 3m 4.8 (1.4) vs 6m 4.8 (1.4)	
		**Unscheduled care**	No between group comparison for proportions of emergency department visits and hospitalisation due to asthma.
		*Percentages of reports an emergency department visits due to asthma since the last visit:* Within group comparison from baseline, 3mo and 6mo; %	‘There was no difference between the PS and AE groups with respect to ED visits for asthma (*P* = 0.51) and hospitalisations for asthma (*P* = 0.79).
		I: B 4.3 vs 3mo 6.2 vs 6mo 7.3	**Consistently shown as no effect.**
		C: B 4.8 vs 3mo 4.2 vs 6mo 3	
		*Percentages of reports on hospitalisation due to asthma since the last visit:* Within group comparison from baseline, 3m and 6m; %	
		I: B 1.8 vs 3mo 2.8 vs 6mo 1.5	
		C: B 3 vs 3mo 2.8 vs 6mo 0.7	
		**Adherence**	No between group comparison for ICS adherence.
		*Inhaled corticosteroid, ICS adherence from data-time record of downloaded data from the ICS monitors:* Within group comparison from baseline, 3m and 6m; mean (SD)	
		I: B 61 (26) vs 3mo 58 (28) vs 6mo 55 (29)	
		C: B 61 (28) vs 3mo 53 (27) vs 6mo 52 (28)	**Insufficient details to gauge effectiveness.**
**Macy 2011 [**24**]**			
US, RCT, FU: 5 weeks, one centre, carers of 86 children, majority White American; age more than 19 y-old, proportion with limited health literacy, n (%) 27 (31%), baseline asthma control: Mild.	Participants (carer of child with asthma) received video-based asthma education materials after receiving care at the emergency department.	**Asthma control**	No relevant outcome.
**The overall risk of bias: High risk**	Groups	*Unscheduled care	There is a significant difference between the proportion of parents with limited and adequate health literacy within the control group in terms of visits to PCP and ED visits. However, the difference between intervention and control is not mentioned.
	I: video-based asthma education material	*Healthcare utilisation at 5-week follow-up:*	**Insufficient details to gauge effectiveness.**
	C: written asthma education material	Within group comparison of return visit to the primary care practitioners (PCP) between low & adequate health literacy carers; n(%)	
	*AHL-adequate health literacy	I: LHL 71.4 vs AHL 57.1, *P* = 0.5	
	*LHL-limited health literacy	C: LHL 23.1 vs AHL 67.7, *P* = 0.009	
		*Healthcare utilisation at 5-week follow-up:*	
		Within group comparison of return visit to the emergency department (ED) between low & adequate health literacy carers; n (%)	
		I: LHL vs AHL 57.1, *P* = 0.5	
		C: LHL 23.1 vs AHL 67.7, *P* = 0.009	
		**Perceived sense of asthma control**	No between group comparison. Perceived sense of asthma control of both groups remained unchanged at follow-up.
		*Perceived sense of asthma control at baseline and at 5-week follow-up:* Both group comparison between low & adequate health literacy carers; median (IQR)	**Insufficient details to gauge effectiveness.**
		LHL: 29(27.3) vs AHL:30(28.3), *P* = 0.45	
		**Knowledge**	No between group comparison. “Improvement in asthma knowledge at follow-up was realized for low-literacy parents regardless of the type of educational intervention with low HL at follow-up was significant” *P* < 0.0001.
		*Change in asthma knowledge score at baseline:* Both group comparison between low & adequate health literacy carers; %	**Consistently shown as no effect.**
		LHL: 33.3 vs AHL: 59.3, *P* = 0.025	

mo – month, RCT – randomized controlled trial, SD – standard deviation, MD – median, y – year, QoL – quality of life

***Participants characteristics*:** The three US studies included majority and minority populations [24,27,28]. Yin et al. (2017) included mainly Latin Americans (Hispanics); Apter et al. (2011) included mainly African-Americans, and the majority of the population in the study by Macy et al. (2011) was White American. The trial conducted in Canada by Poureslami et al. (2012) included participants from minority Chinese and Punjabi ethnic groups [25]. The study conducted in Turkey by Ozyigit et al. (2014) did not specify the ethnicity of the population [26]. Participants’ asthma status was described as uncontrolled [26,28]; mild intermittent, persistent or moderate-severe asthma [27]; mild asthma [24]. One study did not describe the participants’ level of asthma control [25].

***Study setting***: Two studies were conducted in primary care settings [26,28]. Three studies were conducted in secondary/tertiary care settings (specialist paediatric [27] or emergency department [24], university-based pulmonary medicine clinic [25]).

***Geographical area and socioeconomic status***: Four studies were described as set in an urban environment [24,25,27,28]; three described their population as of low socioeconomic status [24,27,28], the fourth had less than a third in the ‘working-class group’ [25]. The non-urban study described the population as living in the most socio-economically under-developed province in the country [26].

***Health literacy status of the population:*** Only three studies measured the level of health literacy of their participants. One study, which used the validated Newest Vital Sign (NVS), estimated that 70% of the study population had limited health literacy level [27]. Two other studies measured the health literacy level of the study population using the Short Test of Functional Health Literacy in Adults (sTOFHLA) (stating that the mean reading comprehension score was ‘adequate’[28]) or the Rapid Estimate of Adult Literacy in Medicine (REALM) (reporting that ‘two-thirds of the study population had an ‘adequate’ level of health literacy’) [24]. Two studies included ‘immigrants’ [25] or ‘illiterates’ [26] as their study population.

***Intervention characteristics:*** **Table 4** summarises the interventions (see detailed descriptions in Table S4 in the **Online Supplementary Document**). All studies had one intervention and one control group [24,26-28] except Poureslami [25], which had three intervention groups [25].

All interventions included education delivered through various methods; one used a face-to-face personalised problem-solving approach [28], two used video-based education [24,25], and two used education with pictorial asthma action plans [26,27] although only one of these explicitly tailored its action plan to low-literacy level [27]. Three interventions were delivered by research assistants [26-28] and one by a respiratory physician [26]. Four studies specified the language used to deliver the intervention; English or Spanish [27,28], ‘native language’ [26], Punjabi or Mandarin [25]. Only two studies specified the duration of the intervention: 20-minute video [24] or four 30-minute problem-solving sessions [28]. Length of follow-up ranged from five weeks to a year [24-26,28]. One study assessed the immediate understanding of a pictorial asthma action plan [27] rather than longer-term outcomes

### Quality assessment of the included studies

**Figure 3:**
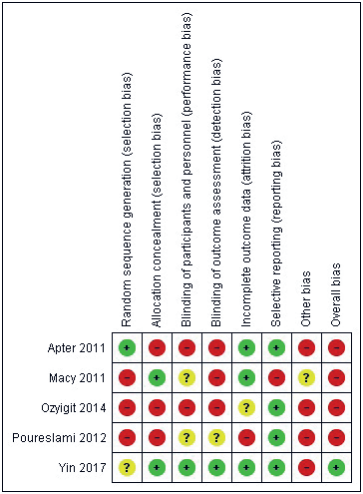
Risk of bias summary: judgement about each risk of bias item for each included study. Green – low risk, red – high risk, yellow – unclear.

Only one study was at an overall low risk of bias [27] (**Figure 3**). The high risk of bias in the other four studies was typically due to no description of random sequence generation or blinding of outcome assessment. Other biases included no specified sample size [26,28] and use of non-validated tools to measure outcomes [25].

### Quality of description and replication

All the studies described the rationale for the essential elements included in the intervention, but none were explicitly guided by a theoretical framework. Three studies lacked descriptions of how the intervention was provided:[24,25,27] for example; one report was unclear whether the video-based intervention was provided individually or in a group [24]. Brief descriptions of the interventions are in **Table 4**; (see detailed descriptions in Table S4 in the **Online Supplementary Document**).

### Effectiveness of interventions on primary and secondary outcomes

The study at low risk of bias did not report any of our primary outcomes. [27]. Two studies (at high risk of bias) reported health outcomes [26,28], one of which reported a positive outcome for unscheduled care [26]. None of the five studies reported on implementation outcomes (such as uptake/completion of the intervention).

Findings are detailed in **Table 4** and the key points described below.

#### 1) Primary (Health outcomes): Asthma control and unscheduled care

##### Impact on asthma control

Two studies at high risk of bias measured asthma control using validated questionnaires (see **Table 4**) [26,28]. Neither of the interventions had an effect on asthma control.

##### Impact on unscheduled care

Three studies at high risk of bias measured the impact of the intervention on unscheduled care [24,26,28]. One study reduced emergency visits in the intervention group compared to control [26]. One study only reported within-group changes, stating that there was no between-group difference though no statistical comparison was provided [28].

#### 2) Secondary outcomes

##### Impact on knowledge

The low risk of bias study reported a positive outcome on knowledge [27] while the other studies reported no effect [24] (see **Table 4**).

##### Impact on correct inhaler use

A high risk of bias study did not provide sufficient details to gauge the impact of the intervention on correct inhaler use [25].

##### Impact on other practical self-management measures

Other measures included in this review are perceived ease of action plan use, understanding of low-literacy AAP (low risk of bias) [27], perceived sense of asthma control [24], understanding of physician instruction [25] and adherence [28] (high risk of bias). All studies either reported no effect [27,28] or reported insufficient details to gauge effectiveness [24,25] (see **Table 4**).

### Identification of intervention components in relation to the behaviour change

**Figure 4:**
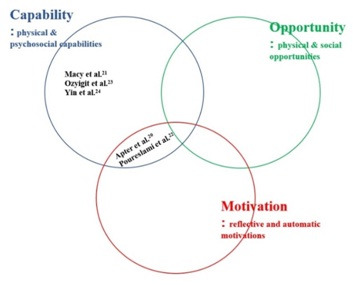
The use of the core components of behaviour in COM-B model in the included studies.

Limited reporting and the lack of effectiveness in the included studies meant that it was not possible to map the components of BCW to effectiveness. The core components of behaviour and the intervention functions used in the included studies based on reported information are provided in **Figure 4**. Reports were sometimes limited: for example, one intervention described providing ‘patient skills’ in its education video [24], with no further description of what was taught.

In terms of the use of the BCW core components of behaviour (COM-B), three studies only addressed ‘capability’ in their interventions [24,26,27]. Two studies, at high risk of bias, addressed a combination of capability and motivation [25,28].

In **Table 3**, we used the published matrix [22] to plot the included studies according to the core components of behaviour change and intervention function. The low risk of bias study used only one intervention function (enablement) [27]. For the high risk of bias studies; two used three intervention functions [25,26], and two studies used two intervention functions [24,28].

Michie et al. (2011) suggest that the core components of behaviour can be linked to the interventions in more than one way. As an example, the use of a pictorial action plan by Ozyigit et al. [26] is ‘education’ as it increases the capability to understand asthma self-management. A pictorial action plan is also a form of ‘enablement’ as it reduces barriers (eg, lack of knowledge/cognitive skills) to self-management of asthma in the event of deterioration. Most of the interventions concentrated on capability components of the behaviour model, and these interventions used functions such as education, training, persuasion and enabling interventions (action plans) to produce behaviour change.

## DISCUSSION

### Summary of findings

This review reports the synthesised findings from five randomised control trials. Four studies, at high risk of bias, concluded that their interventions were ineffective; the only study at low risk of bias did not report on health outcomes. The paucity of studies, limitations in study design and diversity of the interventions meant we are unable to draw conclusions about overall effectiveness on any of our outcomes of interest.

Most studies [24,26-28] included in this review did not describe any theoretical framework underpinning the intervention development, although one conducted prior exploratory work to understand the impact of health literacy in the targeted population [25]. Education, training and enablement are the intervention functions used in these interventions, and the content and the method of delivery varied, including video-based [24,25] and a pictorial action plan [27]. All the interventions used components of behaviour change primarily directed at individuals’ (physical and psychosocial) capabilities; two addressed self-motivation; none targeted opportunity).

### Interpretation of the findings and comparison with previous findings.

#### The use of theory in developing a complex intervention

Health literacy is a complex concept, and as the concept has evolved, a number of definitions have been suggested by researchers and organisations [30]. Tools to measure the health literacy status of populations arise from these definitions and are similarly diverse, making studies in this area heterogeneous and more difficult to interpret. The use of health literacy as a dichotomous variable in many of these tools remained an inherent flaw, especially when health literacy is a spectrum which interacts in complex ways with the environment and socio-cultural factors. In this review, we used a systematically-defined definition by Sørensen et al. (2012) [8] which enabled us to include studies that employed other aspects of health literacy in their intervention, eg, functional health literacy skills [26].

Only one study [25] in our review developed its intervention based on a recognised definition of health literacy (by Nutbeam et al. (2000) [31]). Poureslami et al. (2011), aligned their asthma educational material with the definition of ‘critical health literacy’ which requires sufficient cognitive skills in order to understand, analyse and independently act on adversities in life to care for asthma [29]. In their prior qualitative work, language was found to be a barrier in understanding health information [32]. Thus, in the trial, the education material was delivered using the spoken languages of the participants and was designed to help participants learn and understand beliefs about asthma from the ethno-cultural point of view [25].

Four other studies [24,26-28] did not use specific health literacy definitions, although they used interventional designs which explicitly aimed to improve health literacy (eg, pictograms) as defined by our operational definitions (**Table 1**). None of the studies described any theoretical framework that informed the development of their intervention, implying that the authors had not systematically considered the inter-related barriers among people who struggled with limited health literacy and identified factors which could overcome these barriers.

The Medical Research Council’s framework for developing and evaluating complex interventions clearly outlines the importance of defining a theoretical concept as well as undertaking qualitative exploration [33,34]. A theoretical framework provides a roadmap for the programme of work. In its absence, it is challenging to visualise how the intervention operates to bring about change [35,36]. Interpreting effectiveness is difficult if it is not clear what works and why [33,34].

#### ‘Behaviour Change Wheel’: using a theoretical approach to understand the process of change and to evaluate interventions.

The BCW provides an understanding of what needs to change and how to change it. Targeted behaviour is more likely to change if the specific intervention function is employed. As an example, education using video presentations improved inhaler techniques across the three experimental groups in one study (though the lack of comparison with the control group means it is not possible to gauge effectiveness) [25].

#### A multi-component approach to change behaviour

Previous studies have concluded that the use of more than one strategy in an intervention increased the likelihood of it being effective [10,13]. A review reported that interventions which employed three to four self-management skills were more effective than those using fewer [13]. The five self-management skills considered in that review were problem-solving, taking action, decision making, partnership and resource utilisation [13]. Another review concluded that mixed-strategy interventions focusing on self-management reduced emergency visits, hospitalisations and disease severity in people with long term conditions [10]. Three of the quasi-experimental studies in this review included people with asthma [6,7,37], one of which reduced emergency department visits [6]. Multiple-components in a complex intervention incurs costs in terms of development and manpower [38,39]. However, designing a complex intervention without understanding the behaviour which it aims to change can lead to failure, which is also wasteful. A much criticised example of this is the ineffective UK public health campaign which focused on motivating responsible drinking but failed to reduce opportunity by addressing price and availability [40]. The other point to bring into this section is that the empty marked (†) cells of the matrix (**Table 3**), are gaps that a future multi-component intervention could usefully address.

### Strengths and limitations of this study

We followed Cochrane methodology to search systematically for trials of interventions addressing health literacy in the specific context of asthma self-management. All the stages in the review were duplicated, including the selection of papers, risk of bias assessment and data extraction. Our decision not to search some LMIC-focused databases may mean we missed some relevant studies, though our initial scoping exercise in discussion with a medical librarian suggested this was unlikely. All the included studies were RCTs though we would have accepted other designs of controlled trials. We defined our outcomes with care, ensuring we looked for standardised measures of asthma symptom control and risk of attacks [11] and we included trials based on an evidence-based definition of limited health literacy [8,9].

We used the BCW, a validated framework to describe each of the intervention functions, and interpretation of the findings was conducted by a multidisciplinary team to ensure accuracy. The primary studies have small sample size and diverse in populations which makes it challenging to draw a conclusion from the reported results. Four studies did not use health literacy definitions or framework to map its interventional design. Unfortunately, less than half of interventions in this review reported on asthma control [26,28] or unscheduled care, [24,26,28] limiting the conclusions we could draw. For example, there were insufficient data to present our findings graphically (eg, in a Harvest plot [41]) or to use the GRADE [16] approach to assess the quality of evidence. There was limited description of some of the interventions. We could not, for example be certain whether the ‘patient skills’ described as being included in educational videos in one trial, [24] covered behaviour change techniques such as demonstration of behaviour and/or instruction how to perform the task.

## CONCLUSION

Despite the global importance of the problem, effective interventions addressing health literacy to improve asthma self-management have yet to be developed and evaluated. The studies that we found in this review were diverse, generally at high risk of bias, poorly reported, lacked theoretical underpinning and were ineffective. In designing future interventions, researchers need to be able to identify and understand the factors, including social determinants of health that mediate behaviour change in different contexts (LMICs as well as high-income countries) [38,39]. Tailored asthma self-management interventions for people with limited health literacy should consider a multifaceted approach, including strategies that can be adapted to local needs [39,42], building on theoretical underpinning and careful planning especially in the development stage to optimise effectiveness and sustainability of the intervention.

## Additional material

Online Supplementary Document
