# Supplementary material for: A systematic review of interventions addressing limited health literacy to improve asthma self-management
Source: J Glob Health. 2020 Jun 11;10(1):010427. doi: 10.7189/jogh.10.010428 (PMC7298737; doi:10.7189/jogh.10.010428)
Supplement: Online Supplementary Document [file jogh-10-010428-s001.pdf]

Table S1: GRADE table.

Self-management intervention tailoring to health literacy needs compared with usual care for people with asthma						
**Patient or population: People with asthma**<br>**Settings: Any setting**<br>**Intervention: Self-management intervention tailoring to limited health literacy**<br>**Comparison: usual care**						
**Outcomes**	**Illustrative comparative risks* (95% CI)**		**Relative effect (95% CI)**	**No of Participants (studies involved)**	**Quality of the evidence (GRADE)**	**Comments**
	**Assumed risk**	**Corresponding risk**				
	**Usual care**	**Asthma self-management intervention**				
**Asthma control [20,23]**	See comment	See comment	Not estimable	367 (2)	See comment	The effect is uncertain.<br>These are of high risk of bias studies. Both studies have used different scales, although validated, to measure asthma control and have reported no effects of intervention compared to control [20]. However, one study only reported this in text only [20].
**FEV1 % predicted [20]**	See comment	See comment	Not estimable	333 (1)	See comment	The effect is uncertain.<br>This is of high risk of bias study [20]. It has reported the effect between intervention and control groups, but this is only reported in text only [20].
**Unscheduled care:** *Visits to the emergency department [20,21,23]*	See comment	See comment	Not estimable	496(3)	See comment	The effect is uncertain.<br>These are of high risk of bias studies. One study has reported a positive effect of intervention compared to control [23], one has reported of no effect of an intervention but only mentioned this in text only [20] and one study did not make a comparison between intervention and control groups [21].
**Unscheduled care**: *Visits to the primary care practitioners [21].*	See comment	See comment	Not estimable	129 (1)	See comment	The effect is uncertain.<br>This is of high risk of bias study.[21] It did not report on the effect between intervention group and control but has reported the effect between different health literacy level groups.[21]
**Unscheduled care:** *Hospitalisations due to asthma [20].*	See comment	See comment	Not estimable	333 (1)	See comment	The effect is uncertain.<br>This is of high risk of bias study [20]. It has reported the effect between intervention and control groups, but this is only reported in text only.

Table S2: BCW mapping exercise.

Study	Trial components	Reviewer 1		Reviewer 2		Consensus
		Label & reason	Mapping in matrix<br>1. COM-B<br>2. Intervention function	Label & reason	Mapping in matrix<br>1. COM-B<br>2. Intervention function	Mapping in matrix<br>1. COM-B<br>2. Intervention function

Study	Trial components	Reviewer 1		Reviewer 2		Consensus
		Label & reason	Mapping in matrix<br>1. COM-B<br>2. Intervention function	Label & reason	Mapping in matrix<br>1. COM-B<br>2. Intervention function	Mapping in matrix<br>1. COM-B<br>2. Intervention function
Apter, AJ. et. al (2011) [20]	Pg. 517-518<br>(Methods) Electronic monitor was attached to participants' ICS-containing inhaler and inhaler actuation data were recorded. Subject met with research coordinator to complete four 'problem solving (PS)' sessions facilitated by research coordinators.<br>PS is a four 30-minute session. The individualised intervention has 4 active interactive steps (one per research session).<br>Step 1: This step consists of defining problem particularly in the aspect of adherence. This motivational technique to help the participant view that occurrence of problems (non-adherence) as inevitable, normal and solvable.<br>Step 2: This step consists of brainstorming of alternative solutions.<br>Step 3: This step consists of choosing the best solution by weighing desirable and undesirable consequences.<br>Step 4: The chosen solution is then evaluated and revised.	Capability → Psychological → knowledge or skills; Enablement → increasing means to increase capability beyond education and training and beyond environmental restructuring. Motivation → Reflective → evaluations (beliefs about what is good and bad); Education → Increasing Knowledge or understanding.	COM-B Capability → Psychological → Enablement 1.2 Problem solving [Problem solving]; 9.2 Pros and cons [weighing the consequences, both desirable and undesirable]. COM-B Motivation → Reflective → Persuasion → 2.2 Feedback on Behaviour [data from monitored ICS was shared with the participant in a non-judgmental fashion at each visit].	Capability → Psychological [Skills to engage in the necessary mental process] Enablement → [Provide motivational support for adherence to drugs & the PS sessions provide motivational support that non-adherence as normal & solvable issue] Motivation→ Reflective [PS session allows participants to reflect on ways they can overcome problems with non-adherence, weighing the pro and cons of each solution and evaluate the chosen option.] Education→ [problems with non-adherence were discussed in PS sessions]	COM-B Capability → Psychological Enablement → 1.2 Problem solving COM-B Motivation→ Reflective Education → 2.2 Feedback on behaviour	**1. COM-B Capability: Psychological**<br>**2. Intervention function: Enablement**<br><br>**AND**<br>**3. COM-B Motivation: Reflection**<br>**4. Intervention function: Persuasion**<br><br>**<u>Discussion:</u>**<br>1. The differences<br><br>Although both reviewer 1 and 2 agreed to include the aspect of reflective motivation, both reviewers had difference in opinions on the intervention functions to improve the reflective motivation aspect of the components.<br>2. Consensus<br><br>We agreed that the trial include feedback on behaviour, in this case non-adherence monitored using the actuation of the ICS. Instead of education, the problem-solving session, allows two-way communication of how participants with non-adherence can be persuaded to reflect the problem and work on how to overcome this within their means.

Study	Trial components	Reviewer 1		Reviewer 2		Consensus
		**Label & reason**	**Mapping in matrix**<br>**1. COM-B**<br>**2. Intervention function**	**Label & reason**	**Mapping in matrix**<br>**1. COM-B**<br>**2. Intervention function**	**Mapping in matrix**<br>**1. COM-B**<br>**2. Intervention function**
Macy, L. et. al (2011) [21]	Pg.3<br>(Study protocol) The intervention group viewed a 20-minute asthma educational video entitled "Roxy to the Rescue" before discharge from the ED. This animated program targets urban families with asthmatic children. Key Educational Messages includes: (a) basic facts about asthma, (b) roles of medications, and (c) patient skills.	Capability→ Physical → physical skills; Training→ imparting skills.<br><br>Capability → Psychological→ knowledge or skills; Education → Increasing Knowledge or understanding.	COM-B Capability→ Physical → Training → 4.1 Instruction how to perform behaviour & 6.1 Demonstration of the behaviour (indirectly, film). * [patient skills]. COM-B Capability → Psychological → Education → 5.1 Information about health consequences [basic facts about asthma; roles of medications].	Capability→ Physical [Skills to engage in necessary mental practice] Training → [Imparting patient's skills through video] Capability→ Psychological [Knowledge to engage in necessary mental practice] Education→ [Increasing Knowledge on asthma and medications roles]	COM-B Capability → Physical Training → 4.1 Instruction how to perform behaviour AND 6.1 Demonstration of the behaviour COM-B Capability →Psychological Education → 5.1 Information about health consequences	**1. COM-B Capability: Physical**<br>**2. Intervention function: Training**<br><br>**AND**<br>**1. COM-B Capability: Psychological**<br>**2. Intervention function: Education**<br><br>**<u>Discussion:</u>**<br>We agreed on both of the components of the behaviour change model and the intervention functions for this trial. However, the 'patient skills' component was not described in the report. We, therefore, don't really know whether this covered demonstration of behaviour and/or instruction how to perform behaviour.

Study	Trial components	Reviewer 1		Reviewer 2		Consensus
		**Label & reason**	**Mapping in matrix**<br>**1. COM-B**<br>**2. Intervention function**	**Label & reason**	**Mapping in matrix**<br>**1. COM-B**<br>**2. Intervention function**	**Mapping in matrix**<br>**1. COM-B**<br>**2. Intervention function**
Poureslami, I. et. al (2012) [22]	Pg.543-544<br>(Materials & methods) Three types of interventions: 1) knowledge video - scientific information in terms of asthma symptoms, how to avoid asthma environmental-related and behavioural-related triggers (e.g., exposure to chemicals, dust, pollution, foam, as well as smoking, a sedentary lifestyle, and diet behaviours), and how to manage an asthma attack. Smoking cessation, proper diet, and appropriate exercise for adult asthma patients were also emphasized in the knowledge video 2) community video, community opinions and narratives are used, and<br>covered the community members' cultural beliefs and practices about asthma and its management using social interactive communication styles. In this patient-generated<br>community video, a key informant (an educated elder) addressed the potential misconceptions about asthma management. 3) combination of 1 & 2.	Knowledge video:<br>Capability → Physical → physical skills; Training → imparting skills.<br><br>Capability → Psychological → knowledge or skills; Education → Increasing Knowledge or understanding.<br><br>Community video:<br>Motivation → Reflective > evaluations (beliefs about what is good and bad); Persuasion→ using communication to induce positive or negative feelings to stimulate action	Knowledge video:<br>COM-B Capability → Physical → Training→ 4.1 Instruction how to perform behaviour & 6.1 Demonstration of the behaviour (indirectly, film).* [how to avoid triggers; how to manage an asthma attack].<br>COM-B Capability → Psychological > Education → 5.1 Information about health consequences [asthma symptoms; smoking cessation; diet and exercise].<br>Community video:<br>COM-B Motivation → Reflective → Persuasion → 9.1 Credible source verbal/visual communication in favour of behaviour [key informant (elder) challenged beliefs & practices]	Knowledge video:<br>Capability→ Psychological [Knowledge to engage in necessary mental practice]<br>Training → [Imparting skills]<br>Community video:<br>Motivation → Reflective [reflect on beliefs (what's right & wrong)]<br>Persuasion → [use of community elder to convey the message]<br>Motivation→ Reflective [reflect on beliefs (what's right & wrong)]<br>Coercion → [drawing attention to culture-related misconceptions about asthma]	Knowledge video:<br>COM-B Capability→ Physical<br>Training → 4.1: Instruction on how to perform behaviour AND 6.1 Demonstration of behaviour – video<br>Community video:<br>COM-B Motivation→Reflective<br>Persuasion → 9.1 Credible source<br>COM-B Motivation→Reflective<br>Coercion→ 13.3 Incompatible beliefs	**Knowledge video:**<br>**1. COM-B Capability: Physical**<br>**2. Intervention function: Training**<br>**AND**<br>**1. COM-B Capability: Psychological**<br>**2. Intervention function: Education**<br><br>**Community video:**<br>**1. COM-B Motivation: Reflective**<br>**2. Intervention function: Persuasion**<br><br>**Discussion:**<br>1. The differences<br><br>For knowledge video, reviewer 1 included the combination of increasing psychological capability through education which reviewer 2 had did not. For community video, reviewer 2 included the combination of improvement in reflective motivation through coercion.<br>2. Consensus<br><br>In the knowledge video, the effect of smoking, sedentary lifestyle and eating behaviours, improves one's psychological capability (improving knowledge on health consequences) through education. We agreed to add this aspect for the knowledge video.<br>In the community video, we agreed that persuasion has covered the aspect of communication by asking participants to reflect on beliefs and practices by community elders. Coercion is not suitable, although functions similarly to persuasion, it also to exert mental discomfort or uneasiness by challenging one's beliefs and practices.

Study	Trial components	Reviewer 1		Reviewer 2		Consensus
		**Label & reason**	**Mapping in matrix**<br>**1. COM-B**<br>**2. Intervention function**	**Label & reason**	**Mapping in matrix**<br>**1. COM-B**<br>**2. Intervention function**	**Mapping in matrix**<br>**1. COM-B**<br>**2. Intervention function**
Ozyigit, L. et. al (2014) [23]	Pg.424-426<br>(Methods) The participants were given an interactive asthma education presented by the same, blindfolded respiratory physician, on the same planned day. The education material was prepared on slides including illustrations and the content involved basic mechanism, symptoms of asthma, roles of each medication (symptomatic and preventive medication), administration techniques for any device, signs for exacerbation and environmental control.<br>They were then given the pictorial asthma action plan. The paper-based pictorial asthma action plan has three columns with each column containing instructions with figures for daytime and night-time symptoms.<br>Column 1: Instruction to use of rescue medications in addition to standard treatment when a symptom appears.<br>Column 2: Instruction to take methylprednisolone pills for 5 days and a proton pump inhibitor (prescribed for gastric protection) after information physician when the frequency of the rescue medication increases.<br>Column 3: Instruction to call physician and visit the emergency department, if symptoms persists despite taking the methylprednisolone pills.	Capability → Physical → physical skills; Training → imparting skills.<br><br>Capability → Psychological → knowledge or skills; Education → Increasing Knowledge or understanding.<br><br>Capability → Psychological → knowledge or skills; Enablement → increasing means to increase capability beyond education and training and beyond environmental restructuring.	COM-B Capability → Physical → Training → 4.1 Instruction how to perform behaviour & 6.1 Demonstration of the behaviour (indirectly, illustrations) [Administration techniques for any device].<br>COM-B Capability → Psychological → Education → 5.1 Information about health consequences [symptoms, roles of medications].<br>COM-B Capability → Psychological → Enablement → 1.4 Action planning [taught use of an action plan]	Capability→ Physical [administrative techniques for inhaler devices]<br>Training → [imparting skills].<br>Capability → Psychological [Increasing knowledge on symptoms of asthma, roles of medication, signs of exacerbation & triggers]<br>Enablement → [detail planning in event of daytime & night-time symptoms using action plan]	COM-B Capability → Physical Training → 4.1 Instruction how to perform behaviour AND 6.1 Demonstration of the behaviour<br>COM-B Capability→ Psychological Enablement → 1.4 Action planning	**1. COM-B Capability: Physical**<br>**2. Intervention function: Training**<br><br>**AND**<br>**1. COM-B Capability: Psychological**<br>**2. Intervention function: Enablement**<br><br>**AND**<br>**1. COM-B Capability: Psychological**<br>**2. Intervention function: Education**<br><br>**Discussion:**<br>1. The differences<br><br>Reviewer 1 included the combination of increasing psychological capability through education which reviewer 2 did not.<br>2. Consensus<br><br>We agreed that psychological capability through education function was used to increased knowledge on symptoms of asthma, roles of medications and signs of exacerbations & triggers. This combination was added on.

	Trial components	Reviewer 1	Reviewer 2	Consensus

STUDY	POPULATION CHARACTERISTICS

Study	Trial components	Label & reason	Mapping in matrix<br>1. COM-B<br>2. Intervention function	Label & reason	Mapping in matrix<br>1. COM-B<br>2. Intervention function	Mapping in matrix<br>1. COM-B<br>2. Intervention function
Yin HS et. al (2017) [24]<br>Study	Pg. 920-921<br>(Methods)The study intervention was a low-literacy, plain language, pictogram-, and photograph-based asthma action plan. The tool focuses on three areas of chronic asthma management recognized as being confusing for parents/patients, specifically, knowledge of: (1) medications to give, in particular, understanding the need for everyday preventive medications even when the child is sick; (2) need for spacer use to maximize medication delivery to the lungs; and (3) appropriate emergency response (i.e., giving rescue medication right away and seeking medical help). The intervention emphasized these key messages by incorporating specific low-literacy techniques including the use of plain language, pictorial illustrations, photographs, and optimization of design elements (e.g., layout, text design, colour)	Capability → Psychological [knowledge or skills] Enablement → increasing means to increase capability beyond education and training and beyond environmental restructuring.	COM-B Capability → Psychological → Enablement → 1.4 Action planning [Provision of a Low-Literacy AP].	Capability → Psychological [skills to engage in necessary mental practice] Training→ [participants were 'tested' on their knowledge based on the hypothetical AAP]	COM-B Capability→Psychological<br>Training → 8.1 Behavioural practice/rehearsal [Behavioural experiments].	**1. COM-B Capability: Psychological**<br>**2. Intervention function: Enablement**<br><br>**Discussion:**<br>1. The differences<br><br>Reviewer 1 explained that low literacy action plan is a form of enablement and reviewer 2 explained that the hypothetical low literacy action plan is a form of training.<br>2. Consensus<br><br>There was no clear explanation of whether the participants were trained to use the low-literacy action plan. However, we agreed that action plan tailoring to the literacy need is a form an enablement for people with low literacy to use an action plan.

* Not necessarily enough information to confirm

Blue highlight indicates the differences between reviewer 1 and 2

Table S3: Overview of population characteristics of included studies.

	Aim	Ethnicity(main);participant; sample age; sample size (I/C)	SES; Limited HL level; HL tool used	Baseline asthma control	Geographical area; setting
Apter et al., (2011); USA [20]	Investigate the use of a problem-solving (PS) approach to improve medication adherence in patients with moderate or severe asthma	Mixed majority and minority population (African American); patients; more than 18; 333 (165/168)	Low-income: 31.2 ± 7.3; sTOFHLA*	Mean ACQ† score: 1.7 ± 1.1	Urban; primary care and asthma speciality practices
Macy et al., (2011); USA [21]	Compare change in asthma knowledge among parents with low or adequate health literacy after delivery of intervention during child's asthma-related emergency department visit	Mixed majority and minority population (White); parents; more than 19; 129 (62/67)	Low income; 31% (27/86); REALM‡	Pulmonary index score 1§	Urban: Secondary care (emergency department)
Pouresla mi et al.,(2012) ; Canada [22]	Explore the effectiveness of different formats of culturally relevant information and its impact on asthma patients' self-management within the Punjabi, Mandarin, and Cantonese communities	Minority population (Chinese and Punjabi); patients; at least 21 years; 92(22/21/20/22)	Immigrant; -; -	-	Urban; tertiary (university-based pulmonary medicine clinic)
Ozygit et al., (2014); Turkey [23]	Effectiveness of a pictorial asthma action plan on asthma control, health-related quality of life (HRQoL), and asthma morbidity in a population of illiterate women with asthma	Majority ethnic group; patient; 18-55; 34(18/16)	Socio-economically under-developed; -; -	Mean ACT‖ Score: I: 13.95 ± 3.55 Vs C: 13.7 ± 3.25	-; Secondary (primary care hospital outpatient clinic)
Yin et al., (2017); USA [24]	Test the efficacy of a low-literacy, plain language, photograph- and pictogram-based asthma action plan in enhancing parent understanding of child asthma management	Mixed majority and minority population (Hispanic); patient; parents; mean age 35.5 (8.3);217 (109/108)	Low income; 69.9% (151/217); NVS¶	Mild intermittent 31.3%, persistent, 35% and moderate/severe, 33.6%	Urban; Secondary care (two paediatric outpatient clinics);

*sTOFHLA- Short Test of Functional Health Literacy
†ACQ -Asthma Control Questionnaire
‡REALM - Rapid Estimate of Adult *Literacy* in Medicine
§Pulmonary Index Score assess asthma severity (IQR 0-4)
‖ACT – Asthma control Test
¶NVS – Newest Vital Sign

Table S4: Overview of intervention characteristics.

STUDY (YEAR); COUNTRY	INTERVENTION CHARACTERISTICS				
	Intervention description/length	Control/other group description	Procedure; language used	Mode of delivery; length of intervention	Tailored to health literacy level
Apter et al., (2011); USA [20]	Problem-solving (PS) comprised four 30-minute sessions. The individualized intervention involved 4 interactive steps, usually 1 per research session.<br>This first step involved breaking problems into small achievable pieces. The second step was brainstorming for alternative solutions. The third step was choosing the best solution by weighing the consequences, both desirable and undesirable, of each candidate solution. Between the third and fourth meetings, the solution was tried. For the fourth step, the chosen solution was evaluated and revised.<br>As part of this intervention, downloaded data from monitored ICSs were shared with the participant in a non-judgmental fashion at each visit. At these sessions, subjects followed the same PS steps for addressing an additional problem of their own choosing.<br><br>Length: 30-minutes	Asthma Education, like PS, comprised four 30-minute sessions, each about an AE topic unrelated to self-management, adherence, or ICS therapy. The topics covered, 1 at each session, were the following: (1) the proper technique for using an albuterol rescue metered-dose inhaler and a dry powder inhaler or spacer, depending on the patient's medications; (2) the use of peak flow meters; (3) common asthma triggers; and (4) the pathophysiology of asthma. These sessions did not involve discussion of PS or adherence, only didactic presentation of health information.	Questionnaires on sociodemographics, present and past asthma status, and comorbidities were completed. Spirometric results were obtained. Participants estimated their adherence over the last 3 months with the Inhaler Adherence Scale. An electronic monitor was attached to participants' ICS-containing inhaler. Participants were informed that the monitor recorded the time and date of inhaler actuation and that data would be downloaded at each of 8 study visits. Two weeks later (visit 2), subjects were randomized according to a computer-generated algorithm in a 1:1 ratio to either PS or AE. Subjects met with research coordinators monthly for 4 sessions (visits 2-5) of either PS or AE, spirometry, and downloading monitor data. The need for urgent medical care since the last visit was queried. Subjects then continued to meet monthly with research coordinators for 3 additional months (visits 6-8) to download monitor data, but no PS or AE occurred at visits 6 to 8.<br>Language: English & Spanish	Face-to-face<br><br>Length: 6 months	No tailoring was made but PS is a component of health literacy defined by Sorensen.
Macy et al., (2011); USA [21]	The intervention used video-based asthma educational materials. In the intervention group, participants (carer of children with asthma) viewed a video entitled 'Roxy to the	The active-control group received written educational materials to review in the emergency department or at home. The written materials were developed by Michigan Department of Community Health	Subjects were recruited when presenting to emergency department for evaluation of respiratory symptoms in their 2-14 child had physician-diagnosed asthma or history of wheeze. Eligible parents completed orally administered baseline survey (child asthma history, current	The intervention was delivered using video and follow-up was using telephone call<br><br>Length: 5-weeks	Although the intervention was not tailored to health literacy level, an alternative

STUDY (YEAR); COUNTRY	INTERVENTION CHARACTERISTICS				
	Intervention description/length	Control/other group description	Procedure; language used	Mode of delivery; length of intervention	Tailored to health literacy level
	Rescue' before being discharged from the emergency department. The video was developed by the New England Research Institute which targets urban families with asthmatic children. This animated video consists of educational messages including 1) basic facts about asthma 2) roles of medications 3) patients' skills.<br><br>Length: 20 minutes	and are a standard educational material used across Michigan	symptoms, healthcare utilisation, demographic characteristics, parental baseline knowledge and sense of control). After baseline data collection, they were randomised to intervention and control group.<br>The participants were contacted by phone 4-6 weeks after study enrolment. RA was blinded to the type of intervention received by participants and measured knowledge and sense of control during this phone call. Participants will also report on the frequency of child's asthma symptoms and healthcare utilisation.<br>Language: Not specified		method of delivering educational materials was used.
Poureslami et al., (2012); Canada [22]	The intervention involves the development of educational videos using community participatory approach. Two videos (knowledge & community), are culturally and linguistically appropriate as they included people's views and perceptions about health.<br>In the knowledge video, scientific information is given such as asthma symptoms, how to avoid asthma environmental-related and behavioural-related triggers & how to manage an asthma attack. In the community video, community opinions & narratives are used covering the community members' cultural beliefs & practices about asthma and its management using social interactive communication styles. An educated elder addressed potential misconceptions about asthma, provide correct information on how to manage asthma and encourage early intervention.<br><br>Length: not specified	A pamphlet is reversed engineered from the knowledge video with the aim of to use same content but different presentation format.	Eligible subjects are randomly assigned to three experimental groups and view:<br>Grp 1: physician-led knowledge video<br>Grp 2: patient-generated community video<br>Grp 3: Both Videos<br>One comparison group will read:<br>Grp 4: Educational pamphlet<br>Immediately, after reviewing the educational materials, the participants are asked to study questionnaire. Questionnaires include knowledge of asthma symptoms, knowledge of asthma triggers & triggers that could make asthma worse, understanding physician instruction on medication use & proper use of an inhaler. Intervention is done at the 1-month point after the pre-test assessment. Follow-up lasted for 9 months where assessments are done pre-intervention and at 3-month point. Six months post follow up post-test assessment, patients are assessed for their self-management practices<br><br>Language: Punjabi & Mandarin	Video and face-to-face assessment<br><br>Length: 9 months	The educational materials are developed align with health literacy definition by Nutbeam et. al. of critical health literacy where advanced cognitive and social skills are needed to ensure critical analysis of the situation.
Ozygit et al.,(2014); Turkey [23]	The intervention used pictorial asthma action plan (PAAP) which had been previously used among asthma patients with low level of educations. The PAAP	The education material was prepared on slides including illustrations and the content involved basic mechanism, symptoms of asthma, roles of each medication	Patients were alternately assigned to either the study group or the control group according to their admittance order to the department. Both groups were given an interactive asthma education presented by the same, respiratory physician, on	Face-to-face/paper-based asthma action plan<br><br>Length: 6 months	Tailored for the illiterate woman (functional health literacy)

STUDY (YEAR); COUNTRY	INTERVENTION CHARACTERISTICS				
	Intervention description/length	Control/other group description	Procedure; language used	Mode of delivery; length of intervention	Tailored to health literacy level
	has three columns. The first column indicates that, when a symptom appears, the patients should use their rescue medication in addition to their standard treatment. The second column indicates that when the frequency of use of the rescue medication increases, the patient should take methylprednisolone (16 mg tablet 1 2 a day) for 5 days and use a proton pump inhibitor prescribed for the purpose of gastric protection, after informing her physician. The third column indicates that in the presence of symptoms after methylprednisolone treatment, the patients should call their physician and present to an emergency department.<br>PAAP was given on top of education which also given to the control group.<br><br>Length: not specified	(symptomatic and preventive medication), administration techniques for any device, signs for exacerbation and environmental control.	the same planned day.<br>Baseline assessment was conducted including ACT (asthma control test) and SGRQ (St.George Respiratory Questionnaire). The testswere applied to every participant, before the education head-to-head, and at the first, second and sixth-month follow-ups by telephone interviews were done by the same assessor.<br><br>Language: Turkish		
Yin et al., (2017); USA [24]	The intervention is a low-literacy, plain language, pictogram-, and photograph-based asthma action plan. A hypothetical asthma action plan was created involving commonly prescribed medications. The tool focuses on three areas of chronic asthma management recognised as being confusing for parents; 1) medications to give every day even when the child is sick 2) need for a spacer to maximise medication delivery to lungs 3) appropriate emergency response.<br>The intervention emphasised these key messages by incorporating specific low-literacy techniques such as plain language, pictorial	Parents in the control group received standard action plan developed by the American Academy of Allergy Asthma & Immunology. The medication regimen for the hypothetical child, Jason, was typed in. In each zone, medications information is filled in by the provider within a table format. All information is presented in text format.	Parents were randomly assigned to the intervention and control group. Randomisation was done using sealed envelopes in a block of 50 in each. The lead project coordinator is responsible for randomisation procedure but research assistants are blinded to randomisation. Baseline assessments of sociodemographic & asthma related characteristics are collected prior to assessment using written asthma action plans. Action plans are presented to parents concealed within portfolio & instructed not to reveal the action plan to the interviewer.<br>Participants were assessed their knowledge on 1) green zone knowledge 2) Red zone knowledge. Parents are asked to refer to the action plans they were given to answer questions. They are allowed to take as much time as needed to respond.<br><br>Language: English & Spanish	Face-to-face assessment<br><br>Length: Nil	Level of health literacy among parents is not measured quantitatively.<br>However, the intervention was tailored to the low-literacy status

STUDY (YEAR); COUNTRY	INTERVENTION CHARACTERISTICS				
	Intervention description/length	Control/other group description	Procedure; language used	Mode of delivery; length of intervention	Tailored to health literacy level
	illustrations, photographs, and optimisation of design elements.<br><br>Length: not specified				

Table S5: TIDieR Checklist.

Apter, AJ. et. al (2011) [20]

Problem solving to improve adherence and asthma outcomes in urban adults with moderate or severe asthma: a randomized controlled trial.			
Item number	Item	Where located	
		Primary paper (page or appendix number)	Other details
1.	**BRIEF NAME**<br>Provide the name or a phrase that describes the intervention	Pg. 517	(Methods) Individualised problem solving intervention
2.	**WHY**<br>Describe any rationale, theory, or goal of the elements essential to the intervention	Pg. 516	(Abstract) to investigate the use of a problem-solving (PS) approach to improve medication adherence in patients with moderate or severe asthma.
3.	**WHAT**<br>Materials: Describe any physical or informational materials used in intervention delivery or in training of intervention providers.<br>Provide information on where the materials can be accessed (e.g. online appendix, URL)	Pg. 517-518	(Methods) Electronic monitor was attached to participants' inhaled corticosteroid-containing inhaler and inhaler actuation data were recorded. Subject met with research coordinator to complete four 'problem solving (PS)' sessions facilitated by research coordinators. PS is a four 30-minute session. The individualised intervention has 4 active interactive steps (one per research session).<br><br>Step 1: This step consists of defining problem particularly in the aspect of adherence. This motivational technique to help the participant view that occurrence of problems (non-adherence) as inevitable, normal and solvable.<br><br>Step 2: This step consists of brainstorming of alternative solutions.<br><br>Step 3: This step consists of choosing the best solution by weighing desirable and undesirable consequences.<br><br>Step 4: The chosen solution is then evaluated and revised.

Problem solving to improve adherence and asthma outcomes in urban adults with moderate or severe asthma: a randomized controlled trial.			
Item number	Item	Where located	
		Primary paper (page or appendix number)	Other details
4.	Procedures: Describe each of the procedures, activities, and/or processes used in the intervention, including any enabling or support activities	Pg.517	(Methods) Participants were randomized according to a computer-generated algorithm in a 1:1 ratio to either PS or AE (asthma education).<br>Subjects met with research coordinators monthly for 4 sessions (visits 2-5)<br>of either PS or AE, spirometry, and downloading monitor data. The need for<br>urgent medical care since the last visit was queried. Subjects then continued<br>to meet monthly with research coordinators for 3 additional months (visits<br>6-8) to download monitor data, but no PS or AE occurred at visits 6 to 8.
5.	**WHO PROVIDED**<br>For each category of intervention provider (e.g. psychologist, nursing assistant), describe their expertise, background and any specific training given	Pg.518	(Methods) Research coordinators were college<br>graduates interested in health-related or education careers or further schooling who were committed to working with patients and a research experience. They were diverse in race/ethnicity similarly to patients.
6.	**HOW**<br>Describe the modes of delivery (e.g. face-t-face or by some other mechanism such as internet or telephone) of the intervention and whether it was provided individually or in a group	Pg. 517	(Methods) The intervention was delivered via face-to-face and the sessions were done individually.
7.	**WHERE**<br>Describe the type(s) of location(s) where the intervention occurred, including any necessary infrastructure or relevant features	Pg. 517	(Methods) Subjects were recruited from primary care and asthma specialty practices serving low-income inner-city neighbourhoods with a high prevalence of asthma morbidity
8.	**WHEN AND HOW MUCH**<br>Describe the number of times the intervention was delivered and over what period of time including<br>the number of sessions, their schedule,	Pg. 517-518	(Methods) This is a 26-week study. Two weeks after enrolment, participants were randomized to intervention and control group. They were met by the research coordinators monthly for four months to complete 30-minutes 'problem solving' sessions. Upon completing this, on monthly basis, they met the research

Problem solving to improve adherence and asthma outcomes in urban adults with moderate or severe asthma: a randomized controlled trial.			
Item number	Item	Where located	
		Primary paper (page or appendix number)	Other details
	and their duration, intensity or dose.		coordinators to download inhalers' data.
9.	**TAILORING**<br>If the intervention was planned to be personalized, titrated or adapted, then describe what, why, when and how	Pg. 517-518	(Methods) Intervention was delivered according to individual needs and issues with adherence i.e. exploration of own problems during the first session and how to deal with these problems during session 2 and 3.
10.	**MODIFICATIONS**<br>If the intervention was modified during the course of the study, describe the changes (what, why, when, and how).	-	Information not provided
11.	**HOW WELL**<br>Planned: If intervention adherence or fidelity was assessed, describe how and by whom, and if any strategies were used to maintain or improve fidelity, describe them	Pg. 523b	Fidelity to the protocol was then monitored, first by having the researcher-patient interactions observed by project managers in the early stage of the project, and secondly by periodic unannounced observations of visits with participants in the later stages. Procedures and problems were discussed at weekly team meetings with the principal investigators.
12.	Actual: If intervention adherence or fidelity was assessed, describe the extent to which the<br>intervention was delivered as planned.	Pg. 523b	In this way 100% fidelity to the protocol was achieved.

<u>Macy, L. et. al (2011) [22]</u>

Parental health literacy and asthma education delivery during a visit to a community-based pediatric emergency department: a pilot study.			
Item number	Item	Where located	
		Primary paper (page or appendix number)	Other details
1.	**BRIEF NAME**<br>Provide the name or a phrase that describes the intervention	Pg.3	(study protocol) "Roxy to the Rescue" asthma education video
2.	**WHY**<br>Describe any rationale, theory, or goal of the elements essential to the intervention	Pg. 2	To compare change in asthma knowledge among parents with low or adequate health literacy after video or written asthma education delivered during their child's asthma-related emergency department

Parental health literacy and asthma education delivery during a visit to a community-based pediatric emergency department: a pilot study.			
Item number	Item	Where located	
		Primary paper (page or appendix number)	Other details
			(ED) visit
3.	**WHAT**<br>Materials: Describe any physical or informational materials used in intervention delivery or in training of intervention providers.<br>Provide information on where the materials can be accessed (e.g. online appendix, URL)	Pg.3	(Study protocol) The intervention group viewed a 20-minute asthma educational video entitled "Roxy to the Rescue" before discharge from the ED. This animated program targets urban families with asthmatic children. Key Educational Messages includes: (a) basic facts about asthma, (b) roles of medications, and (c) patient skills.
4.	Procedures: Describe each of the procedures, activities, and/or processes used in the intervention, including any enabling or support activities	Pg.3-4	Parents completed an orally administered survey about the child's asthma history, current asthma symptoms, health care utilization, and demographic characteristics. Baseline asthma knowledge and sense of asthma control were assessed. After baseline data collection, parents were randomized to receive either video (intervention) or written (active-control) asthma education materials. Subjects were contacted by telephone, 4 to 6 weeks after study enrolment. At follow-up, the knowledge and sense of control measures were repeated. Subjects also reported on frequency of the child's asthma symptoms and health care utilization.
5.	**WHO PROVIDED**<br>For each category of intervention provider (e.g. psychologist, nursing assistant), describe their expertise, background and any specific training given	-	Information not provided
6.	**HOW**<br>Describe the modes of delivery (e.g. face-t-face or by some other mechanism such as internet or telephone) of the intervention and whether it was provided individually or in a group	Pg. 3	(Study protocol) The intervention was delivered via video. No details provided whether if any member of the research team was present, whether it was given in groups or individual families.
7.	**WHERE**<br>Describe the type(s) of location(s) where the intervention occurred, including any necessary infrastructure or relevant features	Pg. 3	(Study setting & population) Emergency Department of Hurley Medical Centre in Flint, Michigan.
8.	**WHEN AND HOW MUCH**<br>Describe the number of times the	Pg. 3-4	The 20-minute video-based intervention was delivered once at the point of enrolment. Participants were contacted by phone 4 – 6

Parental health literacy and asthma education delivery during a visit to a community-based pediatric emergency department: a pilot study.			
Item number	Item	Where located	
		Primary paper (page or appendix number)	Other details
	intervention was delivered and over what period of time including the number of sessions, their schedule, and their duration, intensity or dose.		weeks later for assessment.
9.	**TAILORING** If the intervention was planned to be personalized, titrated or adapted, then describe what, why, when and how	Pg. 3	(Study protocol) The animated video targets urban families with asthmatic children
10.	**MODIFICATIONS** If the intervention was modified during the course of the study, describe the changes (what, why, when, and how).	-	Information not provided
11.	**HOW WELL** Planned: If intervention adherence or fidelity was assessed, describe how and by whom, and if any strategies were used to maintain or improve fidelity, describe them	-	Information not provided
12.	Actual: If intervention adherence or fidelity was assessed, describe the extent to which the intervention was delivered as planned.	-	Information not provided

Poureslami, I. et. al (2012) [23]

Effectiveness of educational interventions on asthma self-management in Punjabi and Chinese asthma patients: a randomized controlled trial			
Item number	Item	Where located	
		Primary paper (page or appendix number)	Other details
1.	**BRIEF NAME** Provide the name or a phrase that describes the intervention	Pg.542	(abstract) Culturally relevant knowledge and community education video intervention.

Effectiveness of educational interventions on asthma self-management in Punjabi and Chinese asthma patients: a randomized controlled trial			
Item number	Item	Where located	
		Primary paper (page or appendix number)	Other details
2.	**WHY**<br>Describe any rationale, theory, or goal of the elements essential to the intervention	Pg.542	(abstract) To explore the effectiveness of different formats of culturally relevant information and its impact on asthma patients' self-management within the Punjabi, Mandarin, and Cantonese communities
3.	**WHAT**<br>Materials: Describe any physical or informational materials used in intervention delivery or in training of intervention providers.<br>Provide information on where the materials can be accessed (e.g. online appendix, URL)	Pg.543-544	(Materials & methods) Three types of interventions: 1) knowledge video - scientific information in terms of asthma symptoms, how to avoid asthma environmental-related and behavioural-related triggers (e.g., exposure to chemicals, dust, pollution, foam, as well as smoking, a sedentary lifestyle, and diet behaviours), and how to manage an asthma attack. Smoking cessation, proper diet, and appropriate exercise for adult asthma patients were also emphasized in the knowledge video 2) community video, community opinions and narratives are used, and covered the community members' cultural beliefs and practices about asthma and its management using social interactive communication styles. In this patient-generated community video, a key informant (an educated elder) addressed the potential misconceptions about asthma management. 3) combination of 1 & 2.
4.	Procedures: Describe each of the procedures, activities, and/or processes used in the intervention, including any enabling or support activities	Pg.545	(Materials & methods) Eligible subjects were randomly assigned to one of the three experimental groups (Group 1 viewed a physician-led knowledge video, Group 2 viewed the patient-generated community video, and Group 3 viewed both the knowledge and community videos) and one comparison group (Group 4), which read an educational pamphlet only.
5.	**WHO PROVIDED**<br>For each category of intervention provider (e.g. psychologist, nursing assistant), describe their expertise, background and any specific training given	Pg. 544	(Materials & methods) The intervention was delivered by community facilitators whose expertise, background and training were not mentioned.
6.	**HOW**<br>Describe the modes of delivery (e.g. face-t-face or by some other mechanism such as internet or telephone) of the intervention and whether it was provided individually or in a group	Pg. 542	(Materials & methods) The intervention lasted for 9 months. This process included an initial pre-test assessment, followed by a 1-month educational intervention (the patient watched the videos or read the pamphlet based on the study group they belonged to), and was then followed by a 3-month follow-up post-test assessment.
7.	**WHERE**	Pg. 543	(Materials & methods) The interventions took place in a convenient

Effectiveness of educational interventions on asthma self-management in Punjabi and Chinese asthma patients: a randomized controlled trial			
Item number	Item	Where located	
		Primary paper (page or appendix number)	Other details
	Describe the type(s) of location(s) where the intervention occurred, including any necessary infrastructure or relevant features		place for the patient—either at their home or in our clinic (university-based pulmonary medicine clinic).
8.	**WHEN AND HOW MUCH**<br>Describe the number of times the intervention was delivered and over what period of time including the number of sessions, their schedule, and their duration, intensity or dose.	Pg. 545	(Materials & methods) We conducted our intervention 1 month immediately after the pre-test, and then had a further follow-up 3 months post-intervention.<br><br>Furthermore, 6 months after the post-intervention, the patients were invited to participate in a telephone follow-up survey to assess their self-reported use of the peak flow meter, whether they followed their action plans, and whether they used their prescribed medications regularly.
9.	**TAILORING**<br>If the intervention was planned to be personalized, titrated or adapted, then describe what, why, when and how	Pg. 543	(Materials & methods) The intervention was developed through community participatory approach. As a result, the videos are culturally and linguistically appropriate.
10.	**MODIFICATIONS**<br>If the intervention was modified during the course of the study, describe the changes (what, why, when, and how).	-	Information not provided
11.	**HOW WELL**<br>Planned: If intervention adherence or fidelity was assessed, describe how and by whom, and if any strategies were used to maintain or improve fidelity, describe them	-	Information not provided
12.	Actual: If intervention adherence or fidelity was assessed, describe the extent to which the<br>intervention was delivered as planned.	-	Information not provided

Ozyigit, L. et. al (2014) [23]

The effectiveness of a pictorial asthma action plan for improving asthma control and the quality of life in illiterate women			
Item number	Item	Where located	
		Primary paper (page or appendix number)	Other details
1.	**BRIEF NAME**<br>Provide the name or a phrase that describes the intervention	Pg.423	(Study protocol) Pictorial asthma action plan
2.	**WHY**<br>Describe any rationale, theory, or goal of the elements essential to the intervention	Pg. 424	(Introduction) To establish the effectiveness of a pictorial asthma action plan on asthma control, health-related quality of life (HRQoL), and asthma morbidity in a population of illiterate women with asthma
3.	**WHAT**<br>Materials: Describe any physical or informational materials used in intervention delivery or in training of intervention providers.<br>Provide information on where the materials can be accessed (e.g. online appendix, URL)	Pg.425-426	(Methods) A pictorial asthma action plan was given to participants and proven to be comprehensible by asthma patients with low education levels. The paper-based pictorial asthma action plan has three columns with each column containing instructions with figures for daytime and night time symptoms.<br><br>Column 1: Instruction to use of rescue medications in addition to standard treatment when a symptom appears.<br><br>Column 2: Instruction to take methylprednisolone pills for 5 days and a proton pump inhibitor (prescribed for gastric protection) after information physician when the frequency of the rescue medication increases.<br>Column 3: Instruction to call physician and visit the emergency department, if symptoms persists despite taking the methylprednisolone pills.
4.	Procedures: Describe each of the procedures, activities, and/or processes used in the intervention, including any enabling or support activities	Pg.424	(Methods) Patients were alternately assigned to either the study group or the control group according to their admittance order to the department. Both groups were given an interactive asthma education presented by the same, blindfolded respiratory physician, on the same planned day. The education material was prepared on slides including illustrations and the content involved basic mechanism, symptoms of asthma, roles of each medication (symptomatic and preventive medication), administration techniques for any device, signs for exacerbation and environmental control.

The effectiveness of a pictorial asthma action plan for improving asthma control and the quality of life in illiterate women			
Item number	Item	Where located	
		Primary paper (page or appendix number)	Other details
			The study group was given, in addition, pictorial asthma action plan. Follow-up interviews were conducted by telephone in the first and second month after initial admission. Patients were invited to the outpatient clinic for evaluation of quality of life and asthma control after six months including assessment pf non-scheduled hospital or emergency visits and clinical measures
5.	**WHO PROVIDED**<br>For each category of intervention provider (e.g. psychologist, nursing assistant), describe their expertise, background and any specific training given	Pg.424	(Methods) Blindfolded respiratory physician.
6.	**HOW**<br>Describe the modes of delivery (e.g. face-t-face or by some other mechanism such as internet or telephone) of the intervention and whether it was provided individually or in a group	Pg. 424	(Methods) The intervention was delivered via face-to-face.<br><br>No information provided whether the intervention was delivered individually or in groups.
7.	**WHERE**<br>Describe the type(s) of location(s) where the intervention occurred, including any necessary infrastructure or relevant features	Pg. 424	(Methods) Primary care hospital outpatient clinic
8.	**WHEN AND HOW MUCH**<br>Describe the number of times the intervention was delivered and over what period of time including the number of sessions, their schedule, and their duration, intensity or dose.	Pg. 424-425	(Methods) The intervention was delivered at the point of enrolment.<br><br>No data provided on how long it takes to teach participants the use of pictorial action plan.
9.	**TAILORING**<br>If the intervention was planned to be personalized, titrated or adapted, then describe what, why, when and how	Pg. 425-426	(Methods) The pictorial asthma action plan was design to be easily comprehensible by asthma patients with low level of education.
10.	**MODIFICATIONS**<br>If the intervention was modified during the	-	Information not provided

The effectiveness of a pictorial asthma action plan for improving asthma control and the quality of life in illiterate women			
Item number	Item	Where located	
		Primary paper (page or appendix number)	Other details
	course of the study, describe the changes (what, why, when, and how).		
11.	**HOW WELL**<br>Planned: If intervention adherence or fidelity was assessed, describe how and by whom, and if any strategies were used to maintain or improve fidelity, describe them	-	Information not provided
12.	Actual: If intervention adherence or fidelity was assessed, describe the extent to which the<br>intervention was delivered as planned.	-	Information not provided

Yin HS et. al (2017) [24]

Use of a low-literacy written action plan to improve parent understanding of pediatric asthma management: A randomized controlled study.			
Item number	Item	Where located	
		Primary paper (page or appendix number)	Other details
1.	**BRIEF NAME**<br>Provide the name or a phrase that describes the intervention	Pg. 919	(Abstract) Low-literacy asthma action plan
2.	**WHY**<br>Describe any rationale, theory, or goal of the elements essential to the intervention	Pg. 920	(Introduction)To test the efficacy of a low-literacy, plain language, photograph- and pictogram-based asthma action plan in enhancing parent understanding of child asthma management
3.	**WHAT**<br>Materials: Describe any physical or informational materials used in intervention delivery or in training of intervention providers.<br>Provide information on where the materials can be accessed (e.g. online appendix, URL)	Pg. 920-921	(Methods)The study intervention was a low-literacy, plain language, pictogram-, and photograph-based asthma action plan.<br><br>The tool focuses on three areas of chronic asthma management recognized as being confusing for parents/patients, specifically, knowledge of: (1) medications to give, in particular, understanding the need for everyday preventive medications even when the child is sick; (2) need for spacer use to maximize medication delivery to the lungs; and (3) appropriate emergency response (i.e., giving rescue medication right away and seeking medical help). The intervention

Use of a low-literacy written action plan to improve parent understanding of pediatric asthma management: A randomized controlled study.			
Item number	Item	Where located	
		Primary paper (page or appendix number)	Other details
			emphasized these key messages by incorporating specific low-literacy<br>techniques including the use of plain language, pictorial illustrations, photographs, and optimization of design elements (e.g., layout, text design, colour)
4.	Procedures: Describe each of the procedures, activities, and/or processes used in the intervention, including any enabling or support activities	Pg. 920	(Methods) Parents with children with asthma were enrolled from 2 sites. Parents give consent for participations. Interviews were conducted in Spanish/English according to preference of parents. Eligible parents were randomised using sealed envelopes arranged in blocks of 50 in each site (random order; 25 intervention, 25 control) Lead project coordinator was responsible for the randomisation but research assistant was blinded to randomisation status. Parents were blinded to randomisation until assessments involving intervention were conducted. Action plans were given in concealed portfolio and were instructed not to reveal the action plan to the interviewer.
5.	**WHO PROVIDED**<br>For each category of intervention provider (e.g. psychologist, nursing assistant), describe their expertise, background and any specific training given	Pg. 920	(Methods) Parents were interviewed by research assistant who are blinded to randomization status of parents. Expertise, background and training for intervention provider was not mentioned
6.	**HOW**<br>Describe the modes of delivery (e.g. face-t-face or by some other mechanism such as internet or telephone) of the intervention and whether it was provided individually or in a group	Pg. 920	(Methods) Intervention was given within concealed portfolio and assessment of knowledge of the action plans was done face-to-face.
7.	**WHERE**<br>Describe the type(s) of location(s) where the intervention occurred, including any necessary infrastructure or relevant features	Pg. 920	(Methods) Subjects were enrolled through paediatric outpatient clinics at two sites.
8.	**WHEN AND HOW MUCH**<br>Describe the number of times the intervention was delivered and over what period of time including<br>the number of sessions, their schedule,	Pg. 921	(Methods) At point of assessment, parents were assessed on their knowledge based on the asthma action plan. Parents were asked to refer to the action plan they were given to answer questions and were allowed as much time as they needed to respond to each question.

Use of a low-literacy written action plan to improve parent understanding of pediatric asthma management: A randomized controlled study.			
Item number	Item	Where located	
		Primary paper (page or appendix number)	Other details
	and their duration, intensity or dose.		No other meeting thereafter.
9.	**TAILORING** If the intervention was planned to be personalized, titrated or adapted, then describe what, why, when and how	Pg. 920-921	(Methods) The low-literacy action plan was carefully developed in collaboration with parents and health care providers, with input from individuals with expertise in asthma management, health literacy, and cognitive science.
10.	**MODIFICATIONS** If the intervention was modified during the course of the study, describe the changes (what, why, when, and how).	-	Information not provided
11.	**HOW WELL** Planned: If intervention adherence or fidelity was assessed, describe how and by whom, and if any strategies were used to maintain or improve fidelity, describe them	-	Information not provided
12.	Actual: If intervention adherence or fidelity was assessed, describe the extent to which the intervention was delivered as planned.	-	Information not provided
